# Ecological interactions of sand flies, hosts, and *Leishmania panamensis* in an endemic area of cutaneous leishmaniasis in Colombia

**DOI:** 10.1371/journal.pntd.0011316

**Published:** 2023-05-11

**Authors:** Laura Posada-López, Andrés Velez-Mira, Omar Cantillo, Adriana Castillo-Castañeda, Juan David Ramírez, Eunice A. B. Galati, Fredy Galvis-Ovallos

**Affiliations:** 1 Postgraduate Program in Public Health, School of Public Health, University of São Paulo–USP, São Paulo, Brazil; 2 PECET (Program for the Study and Control of Tropical Diseases) Faculty of Medicine, Universidad de Antioquia, Medellin, Colombia; 3 Centro de Investigaciones en Microbiología y Biotecnología-UR (CIMBIUR), Facultad de Ciencias Naturales, Universidad del Rosario, Bogotá, Colombia; 4 Molecular Microbiology Laboratory, Department of Pathology, Molecular and Cell-based Medicine, Icahn School of Medicine at Mount Sinai, New York City, New York, United States of America; 5 Department of Epidemiology, School of Public Health, University of São Paulo–USP, São Paulo, Brazil; Centro de Pesquisa Gonçalo Moniz-FIOCRUZ/BA, BRAZIL

## Abstract

**Background:**

The transmission dynamics of leishmaniasis are complex. There is also a lack of information about the ecological relationships between the vector/host/parasite at a more local and specific level. The Andean region concentrates more than 50% of Colombia’s cutaneous leishmaniasis (CL) cases. The study of the ecological interactions of sand flies through the identification of blood sources has provided information on the female’s opportunistic behavior, feeding on various hosts. Therefore, this study aimed to determine sand flies’ ecological interactions with *Leishmania* parasites and their blood sources in an endemic area of CL.

**Results:**

A total of 4,621 sand flies were collected, comprising 20 species, in which the most abundant were *Nyssomyia yuilli yuilli* (55.4%), *Psychodopygus ayrozai* (14.5%) and *Ps*. *panamensis* (13.4%). Sequences of 12S gene fragment were analyzed using the BLASTn search tool. Blood-meal source identification was successfully performed for 47 sand flies, detecting seven vertebrate species, human and armadillo being the most frequent. *Leishmania* DNA was amplified in four female pools, constituted by *Ny*. *yuilli yuilli* and *Ps*. *ayrozai*, and the identification through RFLP detected *Leishmania (Viannia) panamensis* in the positive pools.

**Conclusions:**

The interactions between the sand fly species, local mammalian fauna and the *Leishmania* parasite in this active focus of CL, provide evidence of the potential role of two different species in the maintenance of the parasite transmission, important information for the understanding of the ecoepidemiology and transmission dynamics of the disease in Andean endemic areas. However its necessary further evaluations of the vector and host competence in the transmission and maintenance of *Leishmania* spp, in these complex and diverse areas.

## Author summary

The transmission dynamics of leishmaniasis are complex due to the variety of sand fly species, parasites and hosts involved. Although Colombia is considered an endemic country for the disease, the biology, ecology, and identification of blood-feeding sources of sand flies in some endemic areas remain unexplored. For this reason, this study aimed to analyze parameters related to the ecological interactions of the elements of the transmission cycle of cutaneous leishmaniasis. Interactions were detected through parasite and vertebrate DNA identification in sand flies collected intra- and peridomiciliary. Our findings show relevant information on distinct transmission patterns and the participation of different sand fly species in the maintenance of *Leishmania panamensis* in those environments, suggesting *Nyssomyia yuilli yuilli* as its primary vector and *Psychodopygus ayrozai* as a bridging species between wild and peridomiciliary cycle.

## 1. Introduction

Leishmaniases are caused by trypanosomatids of the genus *Leishmania*, and approximately 20 species can cause different clinical manifestations in humans, including cutaneous, mucocutaneous, and visceral leishmaniasis [1–3]. This disease is endemic in 98 countries, and according to the World Health Organization (WHO), there are about 0.9–1.6 million new cases per year [4].

In the Americas, between 2001 and 2019, 1,028,054 leishmaniases cases were reported in 17 countries. Colombia presents a high transmission level, having the second highest incidence rate (per 100,000 inhabitants) after Brazil, reporting a total of 5,907 new cases in 2019, of which 98.7% were Cutaneous Leishmaniasis (CL) cases (PAHO/WHO) [5], followed in number by mucosal and visceral cases.

Cutaneous leishmaniasis is a neglected tropical disease, and a special public health problem, since although it does not cause mortality, it has a psychosocial impact on the population [6]. In America, CL has a variety of clinical forms ranging from localized ulcerated lesions, disseminated with papular lesions, diffuse with nodular lesions without ulcers, to mucocutaneous manifestations that mainly affect the nasopharyngeal mucosa [7–9].

In the neotropical region, 21 species of *Leishmania* have been recognized, of which at least 15 cause CL [7]. Colombia is also one of the countries with the highest *Leishmania* parasite diversity, with nine species reported affecting humans. However, CL outbreaks at intra- and peridomiciliary are mainly caused by *L*. *panamensis*, *L*. *braziliensis*, and *L*. *guyanensis* [10–13].

According to the National System of Public Health Surveillance (SIVIGILA for its Spanish acronym), in the department of Caldas, a total of 975 CL cases were reported in the last five years (2017–2021), with an annual average of 195 new cases. Regarding the study of sand flies in this department, 34 species have been recorded [14]. Of those, species such as *Lutzomyia gomezi*, *Lu*. *hartmanni*, *Lu*. *longipalpis*, *Pintomyia ovallesi*, *Pscychodopygus panamensis*, *Nyssomyia trapidoi* and *Ny*. *yuilli yuilli* [15–17], have been indicated as vectors based mainly on criteria such as anthropophilia, distribution coinciding with the disease, and abundance in the focus of transmission. However, there is a lack of studies on the behavior of sand flies in this region, where feeding habits, survival, and competition, among other criteria that lead to vector incrimination are analyzed.

The transmission dynamics of leishmaniases are complex given the high diversity of parasites, potential reservoirs and sand fly species possibly involved in the cycle. Consequently, studies about the ecological interactions of sand flies through the detection of blood sources could provide information on sand flies blood feeding choices [18–22]. Further, this behavior could have an important role in the maintenance of the zoonotic transmission cycle of *Leishmania* [18, 21, 23, 24].

In Colombia, studies on the feeding habit of sand flies have been carried out mainly in visceral leishmaniases foci in the northern part of the country [25–27]. Sandoval et al. studied some interactions between parasite, vector, and host in a CL focus in Eastern Colombia [28]. However, these types of studies in CL foci are scarce in the Andean region. In the department of Caldas, previous studies focused on the distribution and diversity of sand fly species [15–17] without evaluating the role of these species in the transmission dynamics.

From this perspective, this study’s objective was to determine sand flies’ ecological interactions with *Leishmania* parasites and their blood sources (vertebrates in general) in an endemic area of CL. The interactions were investigated through DNA detections of the parasite and the vertebrate in sand flies collected intra- and peridomiciliary.

## 2. Materials and methods

### 2.1 Study area

In the last decade, the department of Caldas had an incidence rate ranging between 11.5 and 37.8 cases per 100,000 inhabitants, with an average annual incidence of 19.7 cases per 100,000 inhabitants (SIVIGILA). The municipality of Victoria presents an average annual incidence of 338.3 per 100,000 inhabitants (2011–2020), with peaks during the years 2011, 2013, and 2018; therefore, it is regarded as an area of intense transmission (PAHO/WHO) [5].

**Fig 1:**
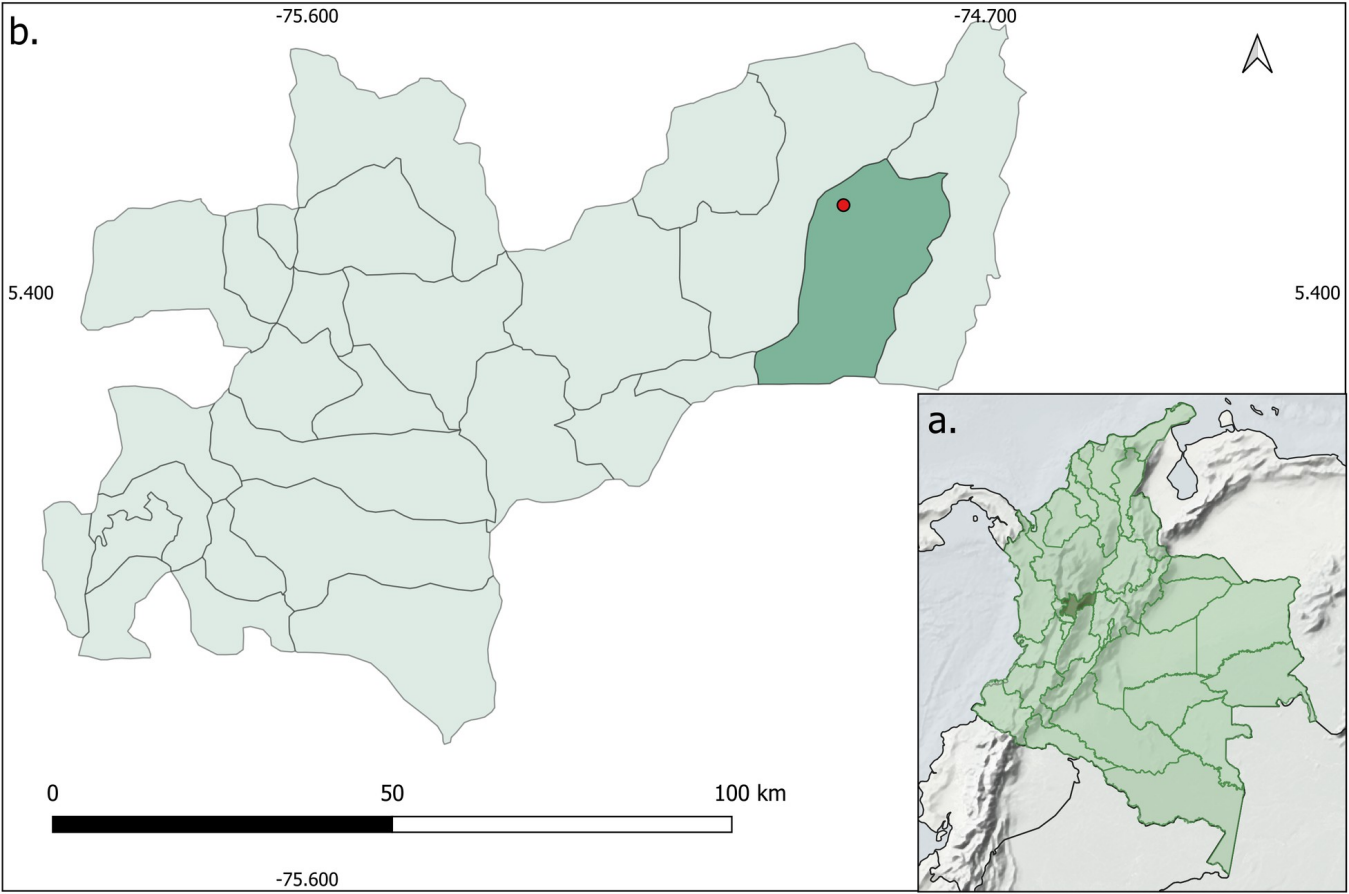
Location of the sand flies’ collection sites. (a) Department of Caldas in Colombia. (b) Municipality of Victoria, red point is the collection site in Carrizales hamlet.

The study was carried out in the municipality of Victoria, in the Carrizales hamlet, located in the East of the department of Caldas (Fig 1). It is part of a subregion known as "*Eje Cafetero*," which is part of the Andean region and is located in the Western and Central Cordilleras. According to the Holdridge life zones system, this region is characterized by tropical moist forests with mean annual temperatures above 24°C, rainfall around 4,000 mm yr−1, and elevations not exceed 1,000 m.a.s.l. [29]. The economy is based on traditional crops, mainly coffee, rubber, cocoa, sugarcane, citrus fruits, avocado, and livestock such as cattle [30]. Carrizales (5°32’8"N, 74°53’26"W) is a hamlet with widely scattered houses around 200 m apart. In a straight line, it is 4.2 km from the nearest urban area and by road 14 km from it. Some houses are very close to the forest (approx. 10 m).

For the captures, six houses were selected. The criteria considered for the selection were the occurrence of autochthonous CL cases during the six months prior to the study’s beginning and the favorable conditions for the development of the sand flies. One of the bedrooms of the dwelling represented the intradomicile. The area around the house, up to 20 m away from it, was considered the peridomicile, having small cocoa and rubber plantations and bordering with paddocks and forest at approx. 10 meters.

### 2.2 Sand flies’ collections and processing

The sand flies’ collections were performed monthly from November 2020 to October 2021 for three consecutive nights. Twelve CDC (Centers for Disease Control and Prevention) automatic light traps were installed in the intra and peridomicile of the six houses, set at 6:00 pm and retrieved at 6:00 am. A Shannon trap was installed in the surrounding forest fragment, 40 m away from House 6, from 6:00 pm–9:00 pm hours, in December 2020, March, April, May, and July 2021, for one night every month. Sand flies were stored in dry tubes for subsequent identification and analysis.

A percentage of the collected females was used to identify natural infection with *Leishmania* and detect blood meal sources. The females were dissected, the head and the last abdominal segments were used for the species morphological identification, and the thorax and first abdominal segments were kept dry at -20°C for molecular biology procedures.

The specimens were identified using taxonomic keys proposed by Galati [31], and the species nomenclature is in accordance with the classification system of the same author. The generic abbreviations follow Marcondes [32].

### 2.3 Molecular analyses: DNA extraction and PCR

After the specific identification of the female specimens and verifying their feeding condition (not engorged), the samples were grouped into pools according to species, date, and place of capture. The engorged females were processed individually.

DNA was extracted using SaMag Tissue DNA extraction kits (Sacace, Italy), which use the SaMag-12 automatic nucleic acid extraction system to extract genomic DNA (Samaga, Cepheid, Italy). The extraction process included lysis, binding, washing, and elution steps. A mechanical maceration step was initially necessary before following the DNA extraction protocol. Extracted DNA was stored at −20°C for amplification by Polymerase Chain Reaction (PCR) method.

#### 2.3.1 Molecular detection of *Leishmania* DNA: PCR and RFLP

A specific *hsp70* gene fragment was amplified to detect the presence of *Leishmania* DNA in the samples, using the PCR-N (593 bp) protocols described by Montalvo et al. [33], employing the primers: F25 (5′-GGACGCCGGCACGATTKCT-3′) and R617 (5′- CGAAGAAGTCCGATACGAGGGA-3′). Negative controls were always included, along with positive controls consisting of 100 fg DNA from *L*. *(Viannia) braziliensis* strain UA301 and *L*. *(V*.*) panamensis* strain UA140.

For *Leishmania* species identification, the positive amplified products from PCR-N were digested simultaneously in a unique tube gathering restriction enzymes RsaI, BsaJI, HindII, and SduI, without prior purification. The Restriction Fragment Length Polymorphism (RFLP-PCR) analysis followed the protocol of Montalvo et al. [34]. Enzymatic digestion of PCR amplicons was performed in a total volume of 20μL. Each digestion contained ×1 optimal buffer (recommended by the manufacturer), 20μL of unpurified PCR product, respectively, and 2U of the enzyme. The reactions were incubated for 3 hours at 37°C (55°C for BsaJI). Restriction patterns were analyzed by electrophoresis in a 4% of agarose gel at 90V.

#### 2.3.2 Blood-meal source identification

A 215-bp fragment of the 12S gene fragment was amplified using primers L1085 (5′-CCC AAA CTG GGA TTA GAT ACC C-3′) and H1259 (5′-GTT TGC TGA AGA TGG CGG TA-3′) as described by Dumonteil et al. [35]. PCR products were purified and submitted for Sanger sequencing. Resulting sequences were edited in SeqTrace software and submitted to BLASTn for similarity search with sequences deposited in GenBank. The high-quality sequences were used to describe the blood-feeding source preferences of the sand flies. Blood meals were inferred using BLASTn against a curated dataset, considering a minimum of 95% identity and an e-value of 10 as a match. Once the probable source of blood (vertebrate) was identified, the geographic distribution of the species in the study area was corroborated.

### 2.5 Analysis of interactions between blood-meal sources, *Leishmania* and sand fly species

Circos plots [36] were used to show the interactions between sand fly species/blood-meal source/*Leishmania* and their frequencies. The analysis of the interactions was done descriptively. An χ2 -test was carried out to calculate significance (p<0.05) and used to compare captures in different environments and the proportion of females of different feeding statuses (engorged/not engorged) for each species. The analyses were carried out using the software PAST version 4.10, and the results were considered significant when p < 0.05. In order to estimate the infection rate in the positive samples for *Leishmania*, the minimum infection rate was calculated according to Paiva et al. [37] with the following formula: Minimum Infection Rate (MIR) = No. of positive groups (pools) × 100/Total of sand flies processed.

## 3. Results

### Sand flies’ fauna and environment distribution

A total of 4,621 sand flies were collected in the sampling (3,511 females and 1,110 males). The specimens belonged to 20 species distributed in 11 genera. The most abundant species in the study area were *Ny*. *yuilli yuilli* (55.4%), *Ps*. *ayrozai* (14.5%), and *Ps*. *panamensis* (13.4%). The other species collected are summarized in Table 1.

**Table 1:** The number of species captured by sex and feeding status of the females. Carrizales hamlet, Victoria, Department of Caldas from November 2020 to October 2021.

Species	Males	Females	Engorged (%)
*Nyssomyia yuilli yuilli*	634	1,926	30 (1.6)
*Psychodopygus panamensis*	113	508	21 (4.1)
*Psychodopygus ayrozai*	180	491	41 (8.4)
*Nyssomyia trapidoi*	21	188	6 (3.2)
*Lutzomyia gomezi*	39	145	7 (4.8)
*Trichopygomyia triramula*	64	106	4 (3.8)
*Lutzomyia bifoliata*	14	49	3 (6.1)
*Lutzomyia hartmanni*	26	45	2 (4.4)
*Psathyromyia shannoni*	2	5	1 (20)
*Brumptomyia* sp.	0	3	1 (33.3)
*Sciopemyia sordellii*	1	17	-
*Psathyromyia barrettoi majuscula*	1	10	-
*Micropygmyia trinidadensis*	5	1	-
*Psathyromyia carpenteri*	1	3	-
*Evandromyia walkeri*	6	4	-
*Evandromyia saulensis*	0	3	-
*Dampfomyia vespertillionis*	0	5	-
*Evandromyia dubitans*	1	2	-
*Psathyromyia dasymera*	1	0	-
*Pressatia camposi*	1	0	-
Total	1,110	3,511	116 (3.3)

Of the 3,511 females screened to determine the feeding status (with blood visible inside their digestive tracts), 116 (3.3%) were engorged. The number and frequency of females engorged by species are shown in Table 1. *Ps*. *ayrozai* was the species with the highest number of engorged females, and together with *Ny*. *yuilli yuilli* and *Ps*. *panamensis* account for 79.3% of the total. The caught engorged females represented 10 of the 20 species collected in the area, and among the predominant species, *Ps*. *ayrozai* showed the highest relative frequency (8.4%) (Table 1). By comparing the percentage of CDC traps-captured engorged females in the intra (2.6%) and peridomicile (2.5%), no significant statistical difference (p>0.01) was observed between the two environments. In the extradomicile, where the captures were made with Shannon traps, the rate of feedings was 17% (Table 2). When intra and peridomicile are compared, *Ny*. *yuilli yuilli* presented the highest relative frequency of engorged females in the intradomicile (statistically significant difference, p<0.05), while *Ps*. *ayrozai* presented the highest proportion of these females in the peridomicile (p<0.05). For *Lu*. *bifoliata*, *Brumptomyia* sp., and *Psathyromyia (Pa*.*) shannoni*, only one engorged female was collected in peridomicile. For the other species, there was no significant statistical difference between the frequency of engorged females in the intra and peridomicile (p>0.05).

**Table 2:** The number of captured and engorged females using CDC traps in intra and peridomicile, and Shannon trap installed extradomicile.

	CDC		Shannon
Species	Intra	Peri	Extra
*Nyssomyia yuilli yuilli*	898 (19)	999 (4)	29 (7)
*Psychodopygus ayrozai*	114 (5)	289 (18)	88 (18)
*Psychodopygus panamensis*	62 (3)	390 (16)	56 (2)
*Nyssomyia trapidoi*	59 (2)	127 (3)	2 (1)
*Trichopygomyia triramula*	45 (1)	60 (3)	1 (0)
*Lutzomyia gomezi*	49 (2)	87 (1)	9 (4)
*Lutzomyia hartmanni*	23 (1)	16 (1)	6 (0)
*Lutzomyia bifoliata*	18 (0)	25 (1)	6 (2)
*Brumptomyia sp*	1 (0)	2 (1)	-
*Psathyromyia shannoni*	2 (0)	3 (1)	-
Total	1,271 (33)	1,998 (49)	197 (34)

### Identification of Feeding sources

Of the 116 females detected with blood at screening, 72 were processed individually to identify the blood source. It was possible to amplify the 12S fragment for blood source detection in 47 samples, representing 65.3% of the total. Of the engorged females, which belonged to ten species, the blood source was characterized in nine of them. In the alignment of the sequences obtained from the positive samples, there was correspondence with several deposited in the GenBank, which allowed the identification of seven species of vertebrates as blood sources (S1 Table). We deposited representative sequences from *Homo sapiens*, *Dasypus novemcinctus*, *Sus scrofa* and *Gallus gallus* to the GenBank database (accession numbers OQ561787, OQ549920, OQ562001, OQ562002).

**Fig 2:**
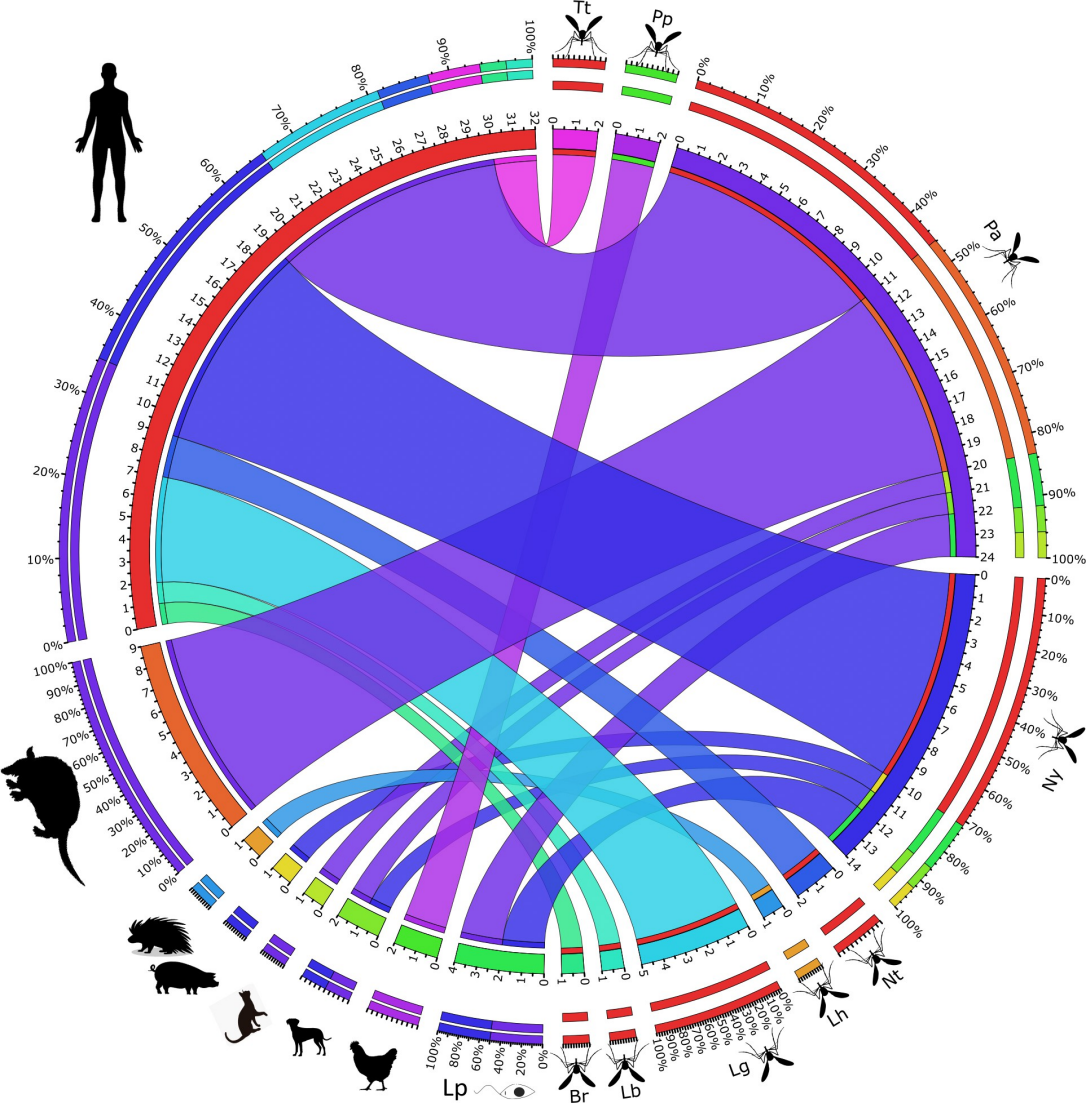
Circos plot of the relationship between blood-meal sources, *Leishmania*, and sand fly species in Carrizales, Victoria (Caldas). Tt, *Triphopygomyia triramula*; Pp, *Psychodopygus panamensis*; Pa, *Ps*. *ayrozai*; Ny, *Nyssomyia yuilli yuilli*; Nt, *Ny*. *trapidoi*; Lh, *Lutzomyia hartmanni*; Lg, *Lu*. *gomezi;* Lb, *Lu*. *bifoliata*: Br, *Brumptomyia* sp; Lp, *Leishmania panamensis*.

Humans were identified as a blood source in most samples, which was detected in seven sand fly species. Other blood sources identified corresponded to domestic animals: dogs, identified in *Ny*. *yuilli yuilli* and *Ps*. *ayrozai* representing (4.3%); chickens in *Ps*. *panamensis* (4.3%); unique samples that matched with cats in *Ps*. *ayrozai*; and pigs in *Ny*. *yuilli yuilli*. Regarding sylvatic animals, porcupines (*Coendou* sp.) were identified in *Lu*. *hartmanni*, and interestingly, 17% of the samples corresponded to armadillos, which were identified only in *Ps*. *ayrozai* (Fig 2).

When analyzing the environmental distribution of the sand fly species and the blood sources identified, it was observed that of the 23 samples found with human blood, 17 (73.9%) were found in the forest. These samples were collected using Shannon traps with a light source and humans as incidental attractants. *Ny*. *yuilli yuilli* and *Ps*. *ayrozai* were the species with the most significant number of interactions with blood meal sources, both species bit humans in all environments. Other blood meal sources, such as dogs in the intradomicile and pigs in the peridomicile, were detected in *Ny*. *yuilli yuilli*.

### Detection and identification of *Leishmania*

A sample of 836 females representing 23.8% of the total captured was analyzed for *Leishmania* DNA detection. These samples were composed of specimens belonging to 15 species and distributed in 323 pools. *Leishmania* DNA was amplified in four of the 323 pools, which corresponded to two of the most common species, *Ny*. *yuilli yuilli* and *Ps*. *ayrozai* (Table 3). Two of these positive samples, one of each species, were processed individually since traces of blood were observed in their abdomen. The other two samples were made up of two females in each pool. Three of the positive pools for *hsp70* were comprised of females captured within the same location (intradomicile of House 2) but at different times (December, April, and October collections).

**Table 3:** Detection of *Leishmania* DNA using *hsp70*, of sand flies collected in Carrizales hamlet, Victoria, Caldas Department from November 2020 to October 2021.

		Females	PCR
Sand fly species	Pools	N	hsp70 (%)
*Nyssomyia yuilli yuilli*	88	347	2 (2.3)
*Psychodopygus ayrozai*	73	173	2 (2.7)
*Psychodopygus panamensis*	51	158	Negative
*Nyssomyia trapidoi*	25	52	Negative
*Lutzomyia gomezi*	31	42	Negative
*Trichopygomyia triramula*	16	24	Negative
*Lutzomyia bifoliata*	17	17	Negative
*Lutzomyia hartmanni*	10	11	Negative
*Brumptomyia* sp.	3	3	Negative
*Evandromyia walker*	3	3	Negative
*Sciopemyia sordellii*	2	2	Negative
*Psathyromyia shannoni*	1	1	Negative
*Dampfomyia vespertillionis*	1	1	Negative
*Psathyromyia barrettoi majuscula*	1	1	Negative
*Psathyromyia carpenteri*	1	1	Negative
Total	323	836	4 (1.2)

The females of one of the pools were captured in the peridomicile of House 1 during April. The *Leishmania* identification by RFLP detected *L*. *(V*.*) panamensis* in the positive pools.

A minimum infection rate (MIR) of 0.5% was estimated, considering the total number of females captured during the study. Regarding the samples by month, the MIR was 1.3% in December, 0.9% in April, and 2.1% in October.

## 4. Discussion

This work provides information on the diversity, ecology, feeding behavior, and natural infection of sand flies, which are key factors in understanding of the epidemiology of CL focus of the Carrizales hamlet. A high diversity (20 species) and low equitability in sand flies were broadly described. The marked dominance of *Ny*. *yuilli yuilli*, *Ps*. *ayrozai*, and *Ps*. *panamensis* represent a potential risk for *Leishmania* parasites transmission.

When comparing the distribution of the engorged females by the environment where these species were collected, a higher relative frequency was observed for *Ny*. *yuilli yuilli* in the intradomicile (80%, Table 2). This result suggests a high degree of anthropophily and an intradomiciliary biting behavior, which is consistent with previous studies that observed the anthropophily and the preference of this species for feeding indoors [38], which could be key factors in the transmission cycle characterization. On the other hand, the low relative frequency of engorged females of this species may be related to a higher activity of nulliparous females in search of a blood source [39], and CDC traps are not very attractive to engorged females given their tendency to move less than unengorged ones [40],

In the peridomicile, the highest frequency of fed females was observed for *Ps*. *ayrozai* (78%). When compared with the intradomicile frequency, the difference was statistically significant (p<0.05), which suggests that this species, although it enters the house, has a peridomiciliary biting habit. Differently, *Ps*. *panamensis* does not show a preference for a particular environment since no difference was found in the frequency of engorged females between intra and peridomicile.

For *Ny*. *yuilli yuilli*, although in this study its primary source was humans (83.3%), other hosts such as domestic dogs and pigs (*Sus scrofa*) were detected, suggesting an opportunistic habit, which is a characteristic that contributes to the vector potential of a species [41].

**Fig 3:**
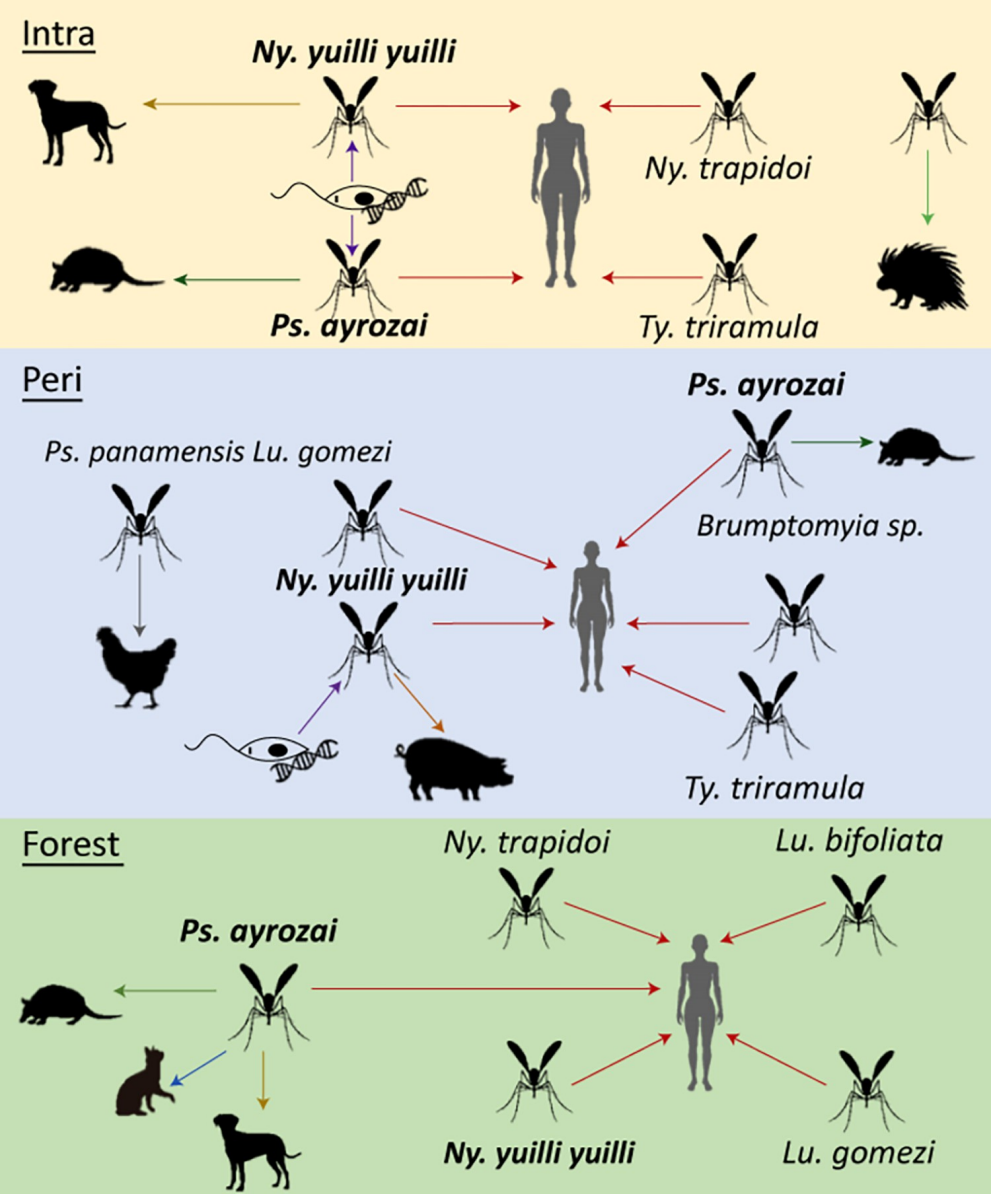
Interactions of sand fly species and blood meal sources in the intra and peridomicile and the forest.

For *Ps*. *ayrozai*, a generalist behavior was also observed. Although humans were the primary blood source (52.4%), a wild host *Dasypus novemcinctus* (armadillo), and domestic animals such as dogs and cats were also identified (Fig 2). The preference of *Ps*. *ayrozai* for *D*. *novemcinctus* was also reported by Le Pont [42] and Rodrigues et al. [20] in French Guyana and the state of Roraima in Brazil, respectively. Here 12S sequences corresponding to the *Dasypus* genus were identified; however, the association with the armadillo species was made based on the distribution reported in the area [43]. This relationship is remarkable, considering armadillos are considered potential reservoirs of *Leishmania spp*, suggesting that *Ps*. *ayrozai* could act as a link species between a wild cycle and a domiciliary cycle (Fig 3).

This study observed a low frequency of dog blood, suggesting that dogs are not very attractive to the sand fly species collected, as previously reported [21, 44]. However, this could also be explained by the diversity of others blood sources available.

Concerning the blood sources in other sand fly species, the anthropophilic habit was detected in seven of the nine species analyzed, representing 68% of the blood sources. Humans were the only source identified in five species, *Lu*. *bifoliata*, *Lu*. *gomezi*, *Ny*. *trapidoi*, *Trychopygomyia triramula* (n = 2) and *Brumptomyia sp*. (n = 1). These results are essential to understand these species’ feeding behavior and propose epidemiological hypotheses about their role in the transmission of *Leishmania* spp.

Nowadays, the feeding habits of *Lu*. *bifoliata* are unknown. It was described as a very anthropophilic species [21]; however, this behavior was dismissed by Alexander et al. due to low frequency in the Shannon trap [17]. In our study, this species appeared sporadically in both intra and peridomicile. Human blood was identified in some females collected in the forest, confirming its anthropophilic behavior.

In the same way, *Ty*. *triramula* has been poorly studied. It has no known anthropophilic behavior and has previously been reported in armadillo burrows [45]. Rigg et al. [24] suggest that it is a generalist species feeding mainly on pigs, cows, dogs, goats, and birds. This species was gathered in all the collections in the present study, corroborating its positive phototropic behavior [46]. However, only one female was found engorged, and human blood was identified as a blood source (S1 Table), indicating a potential opportunistic behavior.

A single blood source was identified in *Lu*. *hartmanni* and *Ps*. *panamensis*, *Coendu* sp., and *Gallus gallus*, respectively. *Lu*. *hartmanni* appeared sporadically in our collections. For *Ps*. *panamensis*, *Gallus gallus* had already been reported as a blood-meal source [24].

A minimal infection rate (IMR) by *Leishmania* was detected in *Ny*. *yuilli yuilli* (0.58%) and *Ps*. *ayrozai* (1.16%). Both species were found in intra and peridomicile and the forest, identifying humans as the primary blood source (Fig 3). It is noteworthy that *Leishmania* DNA was detected in three females collected in house 2, where two cases of CL were recorded during the collection period.

*Nyssomyia yuilli yuilli* has been found with flagellates of *Endotrypanum* in sand flies from the Amazon region in Ecuador [47]. In Colombia, *Ny*. *yuilli yuilli* from the Andean region has already been found infected with *L*. *panamensis* in the department of Boyaca [38]. The present study reports the DNA detection of *L*. *(V*.*) panamensis*, this species, its habit of biting inside the house, and its marked anthropophily.

The detection of *L*. *(V*.*) panamensis* DNA in *Ps*. *ayrozai* is reported for the first time. This sand fly has been associated with armadillo caves and is a potential vector of *L*. *naiffi* [21]. Recent studies recorded females carrying out *L*. *(V*.*) braziliensis* and *L*. *(V*.*) guyanensis* DNA in the municipality of Rio Branco, in the state of Acre/Brazil [48]. This information highlights the need to study this sand fly to evaluate its role as a permissive vector of those *Leishmania* species.

Other sand fly species, including *Lu*. *gomezi*, *Ny*. *trapidoi* and *Ps*. *panamensis* were evaluated but did not show *Leishmania* DNA. However, they are being described as probable vectors of *Leishmania* spp. in other CL foci. *Lu*. *gomezi* is a recognized vector of *L*. *(V*.*) panamensis* in Panamá and Colombia [25, 38, 49] and *L*. *(V*.*) braziliensis* in Venezuela [50]. The importance of this species as a vector is increasing due to its ability to adapt to transformed ecosystems [25]. *Ny*. *trapidoi* is a highly aggressive anthropophilic species and the proven vector of *L*. *(V*.*) panamensis* in Panama and Colombia [51]. *Ps*. *panamensis* has been incriminated as a vector of *L*. *mexicana* [51], and other studies have detected it in Mexico infected with *L*. *infantum* [52] and in Ecuador with *Leishmania* major-like [53]. However, in this study, this species does not seem to play a relevant role in transmission in this focus.

Some limitations must be considered in interpreting the present study, including the convenient sampling and the low number of fed females analyzed to identify the blood-feeding sources. However, our results demonstrate the interactions between the sand fly species, host, and *Leishmania* parasite in the active focus of CL, bringing out evidence of the complexity of the transmission cycle in this region and the necessity for evaluating the role of sand flies in the transmission of *L*. *panamensis*. Additionally, these results contribute to understanding the ecological relations of the main component of the epidemiological triad of Leishmaniasis transmission, which is a crucial element to the development of prevention and control measures on an integrated management vector approach.

Future studies to explore the role of local mammals as possible reservoirs and essays to evaluate the vectorial competence and dispersal of *Ny*. *yuilli yuilli* and *Ps*. *ayrozai* are necessary to understand better the interactions and dynamics of Cutaneous leishmaniasis transmission

## Supporting information

S1 Table Blood meal sources identified from sand fly females collected in Carrizales, Victoria, Colombia. (DOCX)
