# Supplementary material for: Ecological interactions of sand flies, hosts, and Leishmania panamensis in an endemic area of cutaneous leishmaniasis in Colombia
Source: PLoS Negl Trop Dis. 2023 May 11;17(5):e0011316. doi: 10.1371/journal.pntd.0011316 (PMC10204979; doi:10.1371/journal.pntd.0011316)
Supplement: S1 Table — (DOCX) [file pntd.0011316.s001.docx]

| **Sand fly species** | **Blood source (12 S)** | **Accession number** | **E-value** | **Identity** | **Location/Trap** |
| --- | --- | --- | --- | --- | --- |
| *Brumptomyia sp* | *Homo sapiens* | OK058398.1 | 5e-52 | 99,2% | Peri |
| *Lu. bifoliata* | *Homo sapiens* | OM062614.1 | 5e-68 | 98,8% | Extra |
| *Lu. gomezi* | *Homo sapiens* | OM062614.1 | 1e-21 | 98,5% | Extra |
|  | *Homo sapiens* | OM062614.1 | 6e-46 | 99,1% | Extra |
|  | *Homo sapiens* | KC763409.1 | 2e-103 | 99,1% | Extra |
|  | *Homo sapiens* | ON688208.1 | 3e-95 | 98,5% | Extra |
|  | *Homo sapiens* | ON688208.1 | 1e-98 | 100,0% | Peri |
| *Lu. hartmanni* | *Coendou sp* | KX381500.1 | 9e-70 | 95,9% | Intra |
| *Ny. trapidoi* | *Homo sapiens* | OM062614.1 | 4e-27 | 98,7% | Extra |
|  | *Homo sapiens* | ON688208.1 | 8e-106 | 100,0% | Intra |
| *Ny. yuilli yuilli* | *Homo sapiens* | MK069563.1 | 8e-45 | 99,1% | Intra |
|  | *Homo sapiens* | Z57647.1 | 1e-37 | 95,2% | Intra |
|  | *Homo sapiens* | OM062614.1 | 6e-46 | 99,1% | Intra |
|  | *Homo sapiens* | OM062614.1 | 4e-32 | 98,8% | Extra |
|  | *Homo sapiens* | OM062614.1 | 3e-38 | 100,0% | Peri |
|  | *Homo sapiens* | MK069564.1 | 7e-61 | 98,9% | Peri |
|  | *Homo sapiens* | ON688208.1 | 9e-95 | 100,0% | Extra |
|  | *Homo sapiens* | ON688208.1 | 9e-95 | 100,0% | Extra |
|  | *Homo sapiens* | ON688208.1 | 9e-97 | 100,0% | Intra |
|  | *Homo sapiens* | ON688208.1 | 4e-104 | 100,0% | Peri |
|  | *Canis lupus familiaris* | MW487691.1 | 2e-80 | 100,0% | Intra |
|  | *Sus scrofa* | MH603005.1 | 1e-104 | 100,0% | Peri |
| *Ps. ayrozai* | *Homo sapiens* | MT048579.1 | 1e-53 | 97,7% | Intra |
|  | *Homo sapiens* | MT048573.1 | 8e-45 | 98,2% | Extra |
|  | *Homo sapiens* | OM062614.1 | 1e-27 | 98,7% | Extra |
|  | *Homo sapiens* | OM062614.1 | 2e-34 | 98,9% | Peri |
|  | *Homo sapiens* | ON688208.1 | 4e-104 | 99,5% | Extra |
|  | *Homo sapiens* | ON688208.1 | 7e-101 | 100,0% | Extra |
|  | *Homo sapiens* | ON688208.1 | 4e-104 | 100,0% | Extra |
|  | *Homo sapiens* | ON688208.1 | 3e-95 | 100,0% | Extra |
|  | *Homo sapiens* | MT048576.1 | 2e-70 | 100,0% | Extra |
|  | *Homo sapiens* | ON688208.1 | 1e-92 | 100,0% | Extra |
|  | *Homo sapiens* | ON688208.1 | 4e-104 | 100,0% | Peri |
|  | *Dasypus novemcinctus* KX381650.1  *Dasypus novemcinctus* KT818542.1  *Dasypus novemcinctus* KX381650.1 | | 1e-63 | 96,2% | Intra |
|  |  |  | 3e-34 | 95,9% | Extra |
|  |  |  | 3e-39 | 98,0% | Extra |
|  | *Dasypus novemcinctus* | KT818542.1 | 2e-92 | 96,3% | Peri |
|  | *Dasypus novemcinctus* | KT818542.1 | 2e-96 | 98,5% | Intra |
|  | *Dasypus novemcinctus* | KT818542.1 | 2e-97 | 98,1% | Extra |
|  | *Dasypus novemcinctus* | KT818542.1 | 1e-93 | 98,5% | Extra |
|  | *Dasypus novemcinctus* | KT818542.1 | 1e-93 | 99,0% | Intra |
|  | *Canis lupus familiaris* MT622509.1 | | 5e-31 | 98,8% | Extra |
|  | *Felis catus* | KX786344.1 | 3e-80 | 97,8% | Extra |
| *Ps. panamensis* | *Gallus gallus* | OM634640.1 | 2e-102 | 100,0% | Peri |
|  | *Gallus gallus* | OM634640.1 | 7e-91 | 100,0% | Peri |
| *Ty. triramula* | *Homo sapiens* | MT048581.1 | 3e-54 | 98,4% | Peri |
|  | *Homo sapiens* | KF161977.1 | 6e-36 | 95,1% | Intra |
